# Assessment of Genotoxicity and Cytotoxicity of Tepary Bean (*Phaseolus acutifolius*) Seed Protein Extract

**DOI:** 10.3390/life15121937

**Published:** 2025-12-18

**Authors:** Carmen Valadez-Vega, Lizbeth Ortigoza-Fonseca, Gabriel Betanzos-Cabrera, Raúl Velasco-Azorsa, Víctor Manuel Muñoz-Pérez, José A. Morales-González, Belinda Patricia Velázquez-Morales, Aurea Bernardino-Nicanor, Leopoldo González-Cruz, Diego Estrada-Luna, Olivia Lugo-Magaña

**Affiliations:** 1 Área Académica de Medicina, Instituto de Ciencias de la Salud, Universidad Autónoma del Estado de Hidalgo, Ex-Hacienda de la Concepción, Tilcuautla, San Agustín Tlaxiaca 42080, Mexico; 2 Área Académica de Nutrición, Instituto de Ciencias de la Salud, Universidad Autónoma del Estado de Hidalgo, Ex-Hacienda de la Concepción, Tilcuautla, San Agustín Tlaxiaca 42080, Mexico; 3 Académica de Biología, Instituto de Ciencias Básicas e Ingeniería, Universidad Autónoma del Estado del Hidalgo, Ciudad del Conocimiento, Mineral de la Reforma 42184, Mexico; 4 Laboratorio de Medicina de Conservación, Escuela Superior de Medicina, Instituto Politécnico Nacional, Plan de San Luis y Díaz Mirón, Col. Casco de Santo Tomás, Del. Miguel Hidalgo, Ciudad de México 11340, Mexico; 5 Tecnológico Nacional de México/IT Celaya, Antonio García Cubas Pte #600 esq. Av. Tecnológico, Celaya 38010, Mexico; 6 Área Académica de Enfermería, Instituto de Ciencias de la Salud, Universidad Autónoma del Estado de Hidalgo, Ex-Hacienda de la Concepción, Tilcuautla, San Agustín Tlaxiaca 42080, Mexico; 7 Preparatoria Número 1, Universidad Autónoma del Estado de Hidalgo, Av. Benito Juárez S/N, Constitución, Pachuca de Soto 42060, Mexico

**Keywords:** proteins, toxicity, cytotoxicity, genotoxic, *Phaseolus acutifolius*

## Abstract

Beans are widely consumed worldwide and are a good source of amino acids and micronutrients; however, they contain anti-nutrients, such as lectins, tannins, protein inhibitors, saponins, and phytic acid, among others, which can reduce the food’s quality and cause adverse health effects. In this study, we analyzed the genotoxic and cytotoxic effects of a protein extract from *Phaseolus acutifolius* (TBE) seeds. The extract contained some antinutritional compounds, with a higher lectin content and an activity of 2701.85 HU. The acute toxicity test in mice showed that the extract was not lethal at the concentrations tested, as it did not cause any mortality. The in vitro cytotoxicity study on small intestinal epithelial cells indicated that the lectin-rich extract was cytotoxic in both assays, with IC_50_ values of 10.08 µg/mL and 108.91 µg/mL for the free cell and intestinal fragment assays, respectively. In the in vivo study, an erythropoiesis-stimulatory effect was observed, with significant genotoxic damage noted at 48 h, evidenced by 11 micronucleated erythrocytes at 1000 mg/kg TBE. However, no genotoxicity was detected with prolonged treatment times. These results indicate that TBE is cytotoxic within the tested concentration range, and genotoxic damage is influenced by both concentration and exposure time.

## 1. Introduction

Legumes play a significant role in human diets, particularly in developing countries [1]. Preferences for bean consumption vary by region within each country. In Mexico, various domesticated species are consumed depending on the region, including *Phaseolus coccineus* L., *Phaseolus acutifolius* A. Gray, *Phaseolus lunatus* L., and *Phaseolus vulgaris*, with colors such as black, pink, sulfur, pinto, or white. The per capita consumption of beans in Mexico is approximately nine kilograms per year [2].

Beans provide essential nutrients, including protein, vitamin B, minerals, and fiber; they are gluten-free, have a low glycemic index, and exhibit significant antioxidant capacity. Beans are particularly important for populations with limited access to animal protein due to high costs or for those with health conditions that restrict their consumption of animal products, contributing approximately 11% of daily protein intake and 6% of daily energy in the human diet. The bean *Phaseolus acutifolius*, commonly known in northern Mexico as the tepary bean, is a species well-adapted to arid and semi-arid growing conditions. It is a valuable source of nutrients, including protein, carbohydrates, lipids, amino acids, minerals, phenols, and antioxidants. The nutritional significance of the tepary bean lies in its protein content, which varies by variety from 13% to 32% [3,4,5].

Consuming this type of legume provides not only essential nutrients for a balanced diet but also confers physiological benefits that support health. Studies suggest that it may help prevent metabolic diseases such as diabetes, heart disease, and certain types of cancer [6].

Like other legumes, beans contain compounds with anti-nutritional properties that can affect quality by interfering with food digestibility and metabolism, potentially causing physiological harm to consumers. These compounds include lectins, protease inhibitors, tannins, saponins, and phytates [6,7,8].

The quality of a food’s protein is primarily determined by its digestibility, the essential amino acids it provides, and the availability of these amino acids. Despite their excellent nutritional profile, beans have some disadvantages due to anti-nutritional compounds, which significantly reduce protein and amino acid digestibility, thereby affecting nutrient bioavailability [7,9,10].

To minimize potential adverse effects, it is essential to reduce anti-nutritional compounds through processing methods such as thermal extrusion, boiling, pH adjustments, or ultra-high temperature processing [10,11,12,13,14].

Given the potentially toxic effects of certain beans, it is essential to carry out toxicological tests, such as cytotoxicity and genotoxicity assays to assess the potential harm of the compounds or substances present in this food.

One method for assessing potential genotoxic effects is the micronucleus (MN) assay, which detects fragments of chromosomes that are excluded from the nucleus during mitosis [15]. Additionally, performing the MN test on peripheral blood from mice provides a straightforward, rapid, and reliable method to evaluate the cytotoxic effects of the tested sample [16].

The genus *Phaseolus* is a promising source of proteins and peptides with therapeutic potential [17]. Protein isolates derived from beans have been increasingly produced as they offer functional ingredients that enhance food quality. Thus, beans can be utilized to prepare protein isolates rich in essential amino acids and functional compounds, contributing to the nutritional enrichment of products [18].

Given the growing interest in the use of protein isolates and the possible presence of compounds that may pose health risks, it is crucial to evaluate their cytotoxic and genotoxic effects to mitigate any associated hazards. Accordingly, this study aimed to evaluate the cytotoxic and genotoxic effects of a protein extract derived from tepary bean.

## 2. Materials and Methods

### 2.1. Animals

Fifty-nine male CD-1 mice, with a mean weight of 13 g, were used in the micronucleus and median lethal dose (LD_50_) assays. They were obtained from the Institute of Health Sciences-UAEH (Pachuca, Hidalgo, Mexico) and maintained under standard conditions in metallic cages at 23 ± 2 °C and 50 ± 10% humidity, with food (TEKLAD Global 2018S, Harlan, Mexico City, Mexico) and water provided ad libitum on a 12 h light–dark cycle. The animals underwent a one-week acclimatization period before treatments.

Nine male Wistar rats, weighing 200–250 g, were used to obtain intestinal tissue. These animals were also obtained from the Institute of Health Sciences-UAEH and maintained under the same conditions as mice. Prior to the assays, the rats were fasted for 12 h with water provided ad libitum.

### 2.2. Human Erythrocytes

Peripheral blood erythrocytes (Type A) were obtained from a male volunteer donor in accordance with Official Mexican Standards NOM-007-SSA3-2011 [19] and NOM-253-SSA1-2012 [20]. The donor provided signed informed consent, and the study was approved by the Ethics and Research Committee of the Autonomous University of the State of Hidalgo.

### 2.3. Plant Seeds

White seeds of *Phaseolus acutifolius* (tepary bean) used in the experiment were sourced from a local market in Hermosillo, Sonora, Mexico. The seeds were cleaned and stored at 4 °C until use.

### 2.4. Protein Extraction of Phaseolus acutifolius Seeds (TBE)

Proteins were extracted from unprocessed white tepary beans following the methodology proposed by Valadez-Vega et al. [21]. Briefly, a protein solution was prepared by shaking seed powder for 16 h at 4 °C in 10 mM phosphate-buffered saline (PBS), pH 7.4, at a ratio of 1:10 (*w*/*v*). The mixture was then centrifuged at 15,000× *g* for 60 min to obtain the protein extract (TBE). The extract was dialyzed overnight against deionized water at 4 °C, lyophilized, and stored at −20 °C. The protein concentration of TBE was determined using the Bradford method, with bovine serum albumin (Sigma Chemical Co., Burlington, MA, USA) as the standard.

### 2.5. Antinutritional Compounds

#### 2.5.1. Lectins Assay

The serial dilution method was employed [22]. Ninety-six-well microplates were used, with 50 µL of PBS added to each well. A 50 µL sample of the protein solution was serially diluted, and then 50 µL of type A erythrocyte suspension was introduced. The mixture was incubated at room temperature for 1 h, after which the highest dilution that showed hemagglutination was recorded. The results were expressed as hemagglutination activity units per milligram of sample (HAU/mg).

#### 2.5.2. Trypsin Inhibitors Determination

Trypsin inhibitory activity was measured using the synthetic substrate BAPNA (N-benzoyl-DL-arginine p-nitroanilide). The trypsin inhibitor was extracted with NaOH (0.01 N) at pH 9.6 for 2.5 h under stirring, and the supernatant containing the trypsin inhibitors was separated by centrifugation for 30 min at 5000 rpm. A trypsin solution (20 µg/mL in 0.001 N HCl) was added to the supernatant containing trypsin inhibitors and incubated for 10 min at 37 °C. Subsequently, BAPNA (0.4 mg/mL, pH 8.2) (Sigma Chemical Co., St. Louis, MO, USA) was added and allowed to react for 10 min at 37 °C. The reaction was stopped by adding 30% acetic acid, and the absorbance at 410 nm was measured (Perkin Elmer Lambda 40, Waltham, MA, USA). Total trypsin inhibitory activity was expressed as trypsin inhibitor units per milligram of sample (TIU/mg) [23].

#### 2.5.3. Tannins Determination

Tannins were quantified in the protein extract using the modified vanillin–HCl method [21], with (+)-catechin (Sigma Chemical Co., St. Louis, MO, USA) as the reference standard. Briefly, tannins were extracted in methanol for 30 min at room temperature, followed by centrifugation at 3500 rpm. The supernatant was then reacted with vanillin in acidified methanol (0.5:8%), and absorbance was measured at 500 nm using a Perkin Elmer Lambda 40 UV/Vis spectrophotometer. Tannin content was expressed as milligrams of catechin equivalents per gram of sample (mg CE/g).

#### 2.5.4. Saponins Determination

Saponin content was determined following the methodology described by Valadez-Vega et al. [21], with modifications. Briefly, saponins were extracted from the protein extract using a methanol: water solution (85:15%) for 1 h. The solvents were removed by evaporation, and the saponins were diluted in 0.9% NaCl. For saponin identification, the serial dilution method with type A human erythrocytes was employed. Results were expressed as hemolytic units per milligram of sample (HU/mg).

### 2.6. In Vitro Cytotoxicity Assay in Rat Intestinal Epithelial Cells

#### 2.6.1. Intestinal Smear Assay

Four male Wistar rats were fasted for 12 h prior to the experiment. The small intestine was dissected, and 3 cm segments were collected, washed with 0.9% NaCl containing 1.5% streptomycin (Gibco, Grand Island, NY, USA), and inverted on sterile glass rods. The intestinal fragments were then maintained in modified McCoy 5A medium (Gibco, Grand Island, NY, USA), supplemented with 7% fetal bovine serum (FBS; Gibco, Grand Island, NY, USA) and 1.5% streptomycin. The tissue fragments were incubated for 15 min at 37 °C with TBE at concentrations of 0, 5, 10, 50, 100, 200, 500, and 1000 µg/mL, with gentle agitation. Epithelial cell smears were prepared by lightly pressing the intestinal mucosa onto a slide, which was then slid to release the cells. The cells were stained with 0.05% trypan blue (Sigma Chemical Co., Burlington, MA, USA) to assess cell viability using the exclusion technique, where unstained cells were considered viable [24,25]. Half-maximal inhibitory concentration (IC_50_) value was calculated using a nonlinear regression model.

#### 2.6.2. Epithelial Cell Assay

Five male Wistar rats were fasted for 12 h before use. The small intestine was removed, inverted on a glass rod, and washed as described previously. To isolate the epithelial cells, the intestinal fragments were incubated in a 0.25% bovine pancreatic trypsin solution (Fluka Biochemika, Buchs, Switzerland) with constant agitation at 37 °C for 25 min. The cells were then recovered by centrifugation at 2500 rpm, washed three times with modified McCoy 5A medium, and resuspended in the same medium supplemented with 7% FBS and 1.5% streptomycin [3,25].

An aliquot of 600 µL of the cell suspension, containing 8.5 × 10^7^ cells, was transferred to tubes containing 900 µL of the protein extract at concentrations of 0, 1, 2, 5, 10, 50, 100, 200, 500, and 1000 µg/mL, and incubated for 15 min at 37 °C. The cells were then recovered by centrifugation at 2500 rpm and resuspended in modified McCoy 5A medium supplemented with 7% FBS and 1.5% streptomycin. An aliquot of cells was taken and stained with trypan blue to assess cell viability using the exclusion method [25]. The IC_50_ value was determined through a nonlinear regression model.

### 2.7. LD_50_

The experiment was carried out in two phases. In the first phase, 12 male CD-1 mice were divided into four groups and administered TBE intraperitoneally at doses of 0, 10, 100, and 1000 mg/kg. Since no mortality was observed, in the second phase, four groups of three mice were given TBE at doses of 0, 1600, 2900, and 5000 mg/kg to assess animal mortality. The LD_50_ was then calculated [21,26].

### 2.8. Evaluation of the Genotoxic Capacity of TBE by the Micronucleus Assay and Cytotoxicity to Bone Marrow

To assess the genotoxic potential of TBE, 35 male CD-1 mice were divided into five groups and administered single intraperitoneal doses of TBE at 250, 500, and 1000 mg/kg. A negative control group received NaCl solution (0.9%), and a positive control group was treated with daunorubicin (DAU) (Sigma Chemical Co., Burlington, MA, USA) at 3 mg/kg. Peripheral blood samples were collected from the tail of each animal at 0, 24, 48, 72, and 94 h post-treatment. Smears were prepared, fixed with methanol for 5 min, and stained with 4% Giemsa for 20 min [21,27]. To determine the frequency of micronucleated polychromatic erythrocytes (MNPE), 1000 erythrocytes were counted from each sample. Bone marrow cytotoxicity was assessed by scoring 1000 erythrocytes per animal and calculating the ratio of polychromatic erythrocytes to normochromatic erythrocytes (PE/NE ratio). The Ethics and Biosecurity Committee of the Institute of Health Sciences approved the protocol for the micronucleus assay.

### 2.9. Statistical Analysis

ANOVA followed by Tukey’s test (*p* ≥ 0.05) was performed to assess differences between samples and exposure times in the genotoxicity assay, using StatGraphics Centurion 19.1 (StatGraphics, The Plains, VA, USA). IC_50_ values were calculated using SigmaPlot 12.3 (Systat Software Inc., San Jose, CA, USA).

## 3. Results

### 3.1. Protein Extract and Antinutritional Compounds

The protein extract from powder tepary bean, unprocessed, had a concentration of 3.97 mg/mL. Regarding antinutritional compounds, the protein extract exhibited the presence of lectins, trypsin inhibitors, tannins, and saponins, as shown in Table 1.

### 3.2. Epithelial Cell Cytotoxicity

#### 3.2.1. Smear Assay

**Figure 1:**
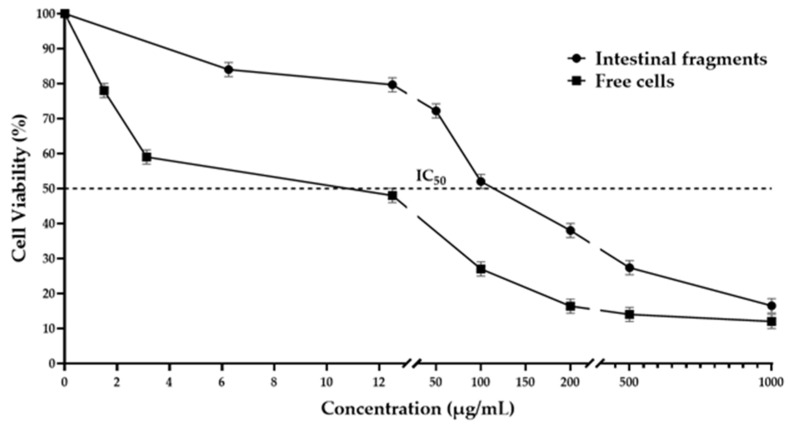
Cytotoxicity of protein extract from *Phaseolus acutifolius* seeds (TBE) in epithelial cells and intestinal fragments. Cells and intestinal fragments were exposed to the indicated concentration of TBE for 15 min. Cell viability was determined as described in the Experimental Section 2.6.1 and Section 2.6.2. The results are presented as the percentage of viable cells relative to untreated cells, which are considered 100% viable. Each value represents the mean ± SD.

Figure 1 shows the cytotoxic effect observed in the epithelial cells from smears prepared from intestinal fragments exposed to TBE, revealing a dose-dependent response. Starting from the lowest dose, a decrease in cell viability was observed, with a continuous reduction noted across all tested doses. The IC_50_ was reached at 108.97 µg/mL, while at the highest dose tested, cell viability was inhibited by 85.3%.

#### 3.2.2. Free Epithelial Cells

The cytotoxic effects of TBE on epithelial cells, isolated from the small intestine, are shown in Figure 1. The results indicate that the extract was toxic to the cells, significantly reducing viability at low concentrations. At 10 µg/mL, the extract inhibited cell viability by 50.2%, with an IC_50_ of 10.08 µg/mL. A consistent reduction in cell viability of approximately 10% was observed at the lower concentrations tested, while the highest concentration (200 µg/mL) produced a marked inhibition of 83.6%. This reduction in viability continued at higher concentrations, with a maximum inhibition of 92.3% at the highest concentration tested.

### 3.3. LD_50_

No lethality was observed in any of the mice at any of the doses tested, indicating that the extract did not induce acute mortality within the evaluated concentration range.

### 3.4. Evaluation of the Genotoxic Capacity of TBE by the Micronucleus Assay and Cytotoxicity to Bone Marrow

**Figure 2:**
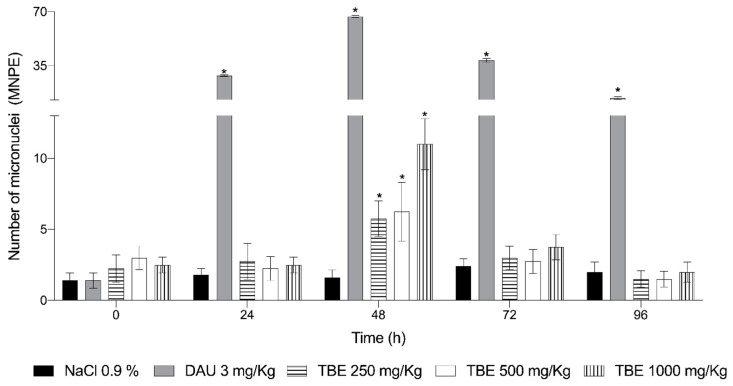
Frequency of micronucleated polychromatic erythrocytes (MNPE) in mice treated with the protein extract from *Phaseolus acutifolius* seeds (TBE) and daunorubicin (DAU). Values represent the mean ± SD of seven mice per group. * indicates a statistically significant difference from the negative control. ANOVA and Tukey tests (*p* ≥ 0.05).

Figure 2 shows the evaluation of the genotoxic effects of TBE, DAU used as a positive control, and physiological saline solution (0.9% NaCl), used as a negative control.

As expected, the mice treated with negative control showed no genotoxicity, with mean values of 1.6, 1.8, 2.4, and 2.0 for the number of MNPE at 24, 48, 72, and 96 h, respectively. In contrast, DAU treatment resulted in a significant increase in MNPE after 24 h, with a mean of 28.6. The strongest genotoxic effect was observed at 48 h, where MNPE levels increased by 33.5-fold compared to the negative control. Genotoxicity persisted up to 96 h, but with a considerable decline in MNPE levels, averaging 14.2, which was 4.7 times lower than the peak observed at 48 h.

Regarding the number of MNPE induced by TBE, genotoxic effects were observed at doses of 250 mg/kg (5.75 MNPE), 500 mg/kg (6.25 MNPE), and 1000 mg/kg (11 MNPE) at 48 h post-administration. At a later time, MNPE counts decreased to the point where no genotoxic damage was detectable.

**Figure 3:**
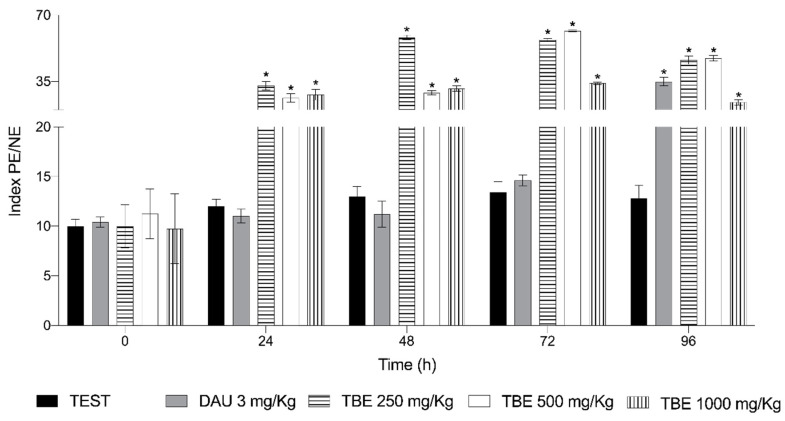
Cytotoxicity of the protein extract from *Phaseolus acutifolius* seeds (TBE). Relationship between the number of polychromatic erythrocytes and the number of normochromatic erythrocytes (PE/NE ratio). Values represent the mean ± SD of seven mice per group. * indicates a statistically significant difference from the negative control. ANOVA and Tukey tests (*p* ≥ 0.05).

Figure 3 shows the results of the cytotoxicity evaluation. DAU treatment led to an increase in the number of PE over time, reaching an average of 34.8 at 96 h. The saline solution had no effect, maintaining an average of 14 PE throughout the study. In contrast, TBE exhibited a stimulatory effect on erythropoiesis beginning at 24 h, with this effect persisting throughout the study. The most significant effect was observed at 48 h with a dose of 250 mg/kg, which was four times greater than the negative control. At 72 h, a dose of 500 mg/kg produced a similar response, 4.2 times greater than that of NaCl.

## 4. Discussion

Beans are a widely consumed legume globally, and in Mexico, they are a staple in the diets of the population [28]. Mexico produces a wide variety of beans, with preferences varying by region. The most commonly cultivated varieties are sulfur, pinto, and black beans, with the latter being predominantly consumed in the central and southern parts of the country. In contrast, lighter-colored varieties, such as the tepary bean (*Phaseolus acutifolius*), are preferred in northern Mexico [2,28].

The protein content in various tepary bean varieties has been reported to range from 18.9% to 21.6% [29]; however, when proteins are extracted using saline solutions, concentrations have been reported between 7.9 and 30.40 mg/mL, with values higher than those observed in this study (3.97 mg/mL).

In comparison to other bean species, the protein concentration in TBE was lower (77.4–85.7%) than that of some other species [30,31], but 74.6% higher than that of mung bean extracts [32]. These differences may be attributed to variations in species, bean variety, and the methods used for protein extraction [3,33].

The presence of antinutritional compounds in grain legumes, such as beans, has been widely reported. Accordingly, their occurrence in protein extracts is expected and is influenced by the extraction method employed. The biological effects of these compounds depend on multiple factors, including their concentration, chemical nature, the species examined, and the physiological status of the animal [34].

Although white tepary bean seeds were used in this study, tannins were detected in TBE; however, their concentration was considerably lower than that previously reported for both white and colored seeds of the same species [3]. It is well established that the content of these polyphenolic compounds is associated with the color of the seed coat, such that more intensely pigmented or darker coats exhibit higher concentration [35]. As the analysis in this study was conducted on a protein extract derived from white seed flour, a low concentration of these compounds was expected.

Saponins were analyzed using the hemolysis method, and their concentration in TBE was found to be higher than that previously reported for protein extracts (0.0161 HU/mg) and flours (0.0078–0.0126 HU/mg) of certain *P. vulgaris* varieties [36]. The differences observed can be attributed to both species-specific variation and the methods employed for protein extraction and saponin determination.

Trypsin inhibitors were detected in the protein extract; although their concentration was low, these antinutritional compounds are known to impair protein digestion. Consequently, their inactivation is essential to prevent adverse effects; this can be achieved through various treatments, including cooking, roasting, extrusion, ultrasound, ultrafiltration, the use of reducing agents, acids or bases, as well as biological processes such as germination and fermentation [3,22,34,37]. In protein extracts from legumes such as beans, lentils, and soybeans, concentrations of these compounds have been reported to range from 11.69 to 89.78 HU/mg, which are substantially higher than those observed in TBE [36]. Moreover, it has been reported that trypsin inhibitors are preferentially extracted under alkaline conditions with NaOH at pH 8.4–10 [34]. Thus, the lower levels observed in TBE compared with other samples can be attributed to the extraction method, since in the present study the protein extract was obtained using PBS at pH 7.4, which likely resulted in the extraction of fewer inhibitors.

Among the proteins found in beans are lectins, which have a globular structure and can be easily extracted with saline solutions [21,33,38]. Lectins are known for their ability to agglutinate erythrocytes, exhibit cytotoxic effects on various cell types [39,40,41,42], and cause adverse effects in laboratory animals [43,44].

For lectin extraction, PBS was used in this study, which proved to be effective for isolating these proteins. A significant concentration of lectins was observed, with an activity of 2701.85 HAU/mg. Previous studies have demonstrated that lectins from different bean species or varieties exhibit varying levels of hemagglutinating activity [44].

In tepary beans, lectin-rich extracts have been reported to have 8618 HAU/mg, while standard bean extracts can show values ranging from 1.5 to 1.9 × 10^10^ HAU/mg [33,45,46]. The differences in hemagglutinating activity are primarily attributed to the source of the lectins and the type of erythrocytes used in the assays. It has been shown that lectin affinity is influenced by the oligosaccharides present on the erythrocyte membrane, as well as the origin of the cells [47]. Since previous studies have employed erythrocytes from different sources, the observed differences in activity are likely due to these variables.

The results of this study indicated a cytotoxic effect of TBE on epithelial cells, both in free cell assays and in intestinal fragments. A slightly greater effect (~10%) was observed in free cells, as indicated by the differences in the IC_50_ values. This disparity may be due to the increased exposure of free cells to the protein extract, whereas cells attached to tissue are somewhat protected from the full effect.

It has been reported that protein extracts from bean seeds can cause damage to the intestines of laboratory animals, resulting in a reduction in the activity of enzymes such as maltase, saccharase, alkaline phosphatase, and hydrolases [48,49]. This damage is thought to be caused by anti-nutritional factors, such as lectins, which are present in high concentrations in several bean varieties. These lectins have been linked to adverse effects, including alterations and abnormal development of the microvilli in the small intestine [24,40].

In vivo studies have demonstrated that feeding laboratory animals beans containing biologically active lectins results in an increase in intestinal size and weight, thickening of the intestinal wall, and alterations in the size, metabolism, and function of the entire digestive tract. Lafont et al. [48] reported that the extent of intestinal damage caused by lectins in laboratory animals is dose-dependent, leading to irregularly sized, shortened, and spaced microvilli, as well as microvilli bleeding and even cell death.

The damage observed in epithelial cells after exposure to TBE can be attributed mainly to the lectins present in the extract, as this is the antinutritional compound found in the highest quantity (2701.85 HAU/mg). Previous studies have reported that tepary beans contain significant concentrations of lectins, which are cytotoxic to malignant colon and cervical cells, as well as to T lymphocytes and 3T3 fibroblasts [25,33,50].

The genotoxicity study revealed that, at 24 h post-treatment, TBE did not induce genotoxic effects at any of the administered doses. However, at 48 h, a significant, dose-dependent increase in the number of MNPE was observed. The animals appeared to recover by 72 h, as indicated by a reduction in the number of MNPE, with no significant difference (*p* ≥ 0.05) compared to the negative control.

Similar results have been reported in studies on laboratory animals, as well as through the Comet and Ames assays. These evaluations demonstrated that seeds from the black and white kidney varieties of *Phaseolus vulgaris*, as well as from the Lady Joy bean, did not cause genotoxic damage, suggesting that these seeds are safe for consumption [50,51,52]. Analysis of human lymphocytes revealed that the aqueous extract of white kidney beans did not induce genotoxicity at the concentrations tested [53]. Additionally, the protein hydrolysate from *Psophocarpus tetragonolobus* (winged bean) seeds, as analyzed by the Ames test, did not induce damage [54].

Furthermore, lectins and protein extracts from other species have exhibited protective actions on genetic material. For example, *Sambucus nigra* lectin has demonstrated DNA-protective and repairing properties [55], while the protein extract from *Spirulina maxima* has shown genoprotective effects in mice [56].

Other natural proteins, such as those derived from *Bauhinia monandra* [57], *Sambucus nigra* [55], *Phaseolus vulgaris* L., *Vigna radiata*, *Arachis hypogaea* L., *Phaseolus vulgaris* L. [42], *Sapindus saponaria* [58], *Microgramma vacciniifolia* [59], have also shown no genotoxic effects.

These effects are often attributed to the ability of certain compounds present in these extracts to trap free radicals, thereby reducing oxidative stress, a process likely mediated by phenolic compounds found in the extracts [42,53]. Some authors have demonstrated that lectins from plant sources can protect and induce DNA repair processes [55,57,60] and prevent the mutagenicity of toxic agents [61]. These processes could be stimulated by lymphokines or cytokines secreted by activated leukocytes [62]. The findings in the present study are consistent with these reports, as significant concentrations of biologically active lectins were found. While these lectins may have caused some cellular damage, their effects were significantly lower than those on genetic material, and were also dose- and time-dependent.

In contrast, proteins derived from various plants have demonstrated dose-dependent genotoxic effects, as observed with those from *Amaranthus hypochondriacus* [21], *Syzygium cumini* [63], *Sapindus saponaria* [58], *Moringa oleifera* Lam. [64], and *Phaseolus coccineus* [18].

The in vivo and in vitro cytotoxicity assays showed that the damage caused by bean proteins was dose-dependent. In the cytotoxicity study in mice, we found that, as visualized by the PE/NE ratio, it was rising, which is an indicator of inhibition in erythrocyte division and maturation [65], indicating that the extract is related to bone marrow damage and cell production [59,66,67].

The highest cytotoxic effect was observed in animals exposed to lower doses of TBE, with the most significant damage at 48 h. Similar results were observed with *Phaseolus coccineus* extracts, where the highest toxicity of the extracts was at 48 h [18]. Regarding the in vitro assay, it was observed that TBE exhibited a dose-dependent effect, with greater cytotoxicity at high doses. This was established as an IC_50_ of 108.97 µg/mL in the study using intestinal fragments, while in free epithelial cells, it was 10.08 µg/m. These results showed that the toxicity of the extract to the cells depends not only on the composition of the extract but also on the protection that the tissue provides to the cells as they are more exposed and susceptible to damage when they are released from the tissue. It is essential to note that, either way, TBE poses a risk, as the consumption of proteins in their bioactive form may cause damage to the intestine.

Studies conducted in both cells and laboratory animals support the findings of our research. In vitro studies demonstrated that protein extracts from various varieties of *Phaseolus acutifolius* and *Phaseolus vulgaris* are cytotoxic to the epithelial cells of the small intestine in rats [3,24,41]. Also, animal studies, in which bean protein extracts were administered, reported that the proteins were toxic to organs, causing changes in intestinal permeability, alterations in the morphology of the microvilli, morphological distortion in the jejunum, alterations in the hydroelectrolytic flow of the intestine, and increased intestinal vascular permeability [68,69].

Other studies involving protein extracts from seeds have indicated that these extracts can be toxic to various cell types, including lymphocytes and intestinal cells [24,54]. Additionally, they can cause damage to organs and even lead to the death of animals when the extracts are administered directly or mixed into their feed [70,71].

The results from our studies provide important insights into the damage caused by protein extracts on intestinal cells and bone marrow, as well as the risks associated with consuming these beans if they are not adequately cooked. It has been reported that protein extracts from seeds may contain anti-nutritional compounds, such as lectins and protease inhibitors, that can cause cellular damage and harm to animals. These compounds have been shown to damage various organs and tissues and may even result in animal death [70,71].

Lectins, a type of protein found in these extracts, have been identified in protein extracts from several bean species, including *Phaseolus vulgaris*, *Phaseolus coccineus*, and *Phaseolus acutifolius* [33,46,49]. The concentration of lectins depends on various factors, including the variety, growing conditions, and extraction methods. Lectins have a potential risk if consumed, as they have been associated with adverse effects, including damage to animal organs, cytotoxicity to both normal and malignant cells, and even death in mice. Therefore, it is crucial to ensure that these proteins are inactivated before consuming these legumes [11,14,46,72].

Additionally, protease inhibitors, such as trypsin inhibitors, are proteins present in seeds and protein extracts. These inhibitors have been linked to several adverse health effects, and it is necessary to inactivate them to prevent harm to consumers [22].

## 5. Conclusions

Our results in the in vivo study indicated that TBE did not cause mortality in mice across the range of concentrations tested. However, it exhibited genotoxic effects, which were most pronounced at 48 h and decreased significantly over time. TBE also showed cytotoxicity to bone marrow, with a stimulatory effect on erythropoiesis. Additionally, in the in vitro study, it caused dose-dependent damage in rat intestinal epithelial cells, with a more pronounced effect observed when the cells were in their free form. The observed toxic effects of TBE are likely attributable to the presence of anti-nutritional compounds, such as lectins, and other substances extracted from it.

Future studies should focus on exploring the specific molecular mechanisms by which lectins and other proteins in TBE exert their toxic effects. This could include research on protein-receptor interactions, cellular signaling pathways, and changes in gene expression in response to exposure. Additional in vivo studies are needed to evaluate long-term toxicity, organ-specific effects, and dose–response relationships. Such research will help establish safe levels and provide critical insights into the safety and potential therapeutic applications of TBE.

## Figures and Tables

**Table 1:** Antinutrient compounds present in TBE.

Lectins (HAU/mg)	Trypsin Inhibitors (TIU/mg)	Tannins (mg CE/g)	Saponins (HU/mg)
2701.85 ± 0.0	6.86 ± 0.55	0.1757 ± 0.0002	20.16 ± 0.0

Antinutrient compounds were quantified in the protein extract from *Phaseolus acutifolius* seeds (TBE). HAU/mg: Hemagglutination activity units/mg of sample, TIU/mg: Trypsin inhibitory units/mg of sample, mg CE/g: mg catechin equivalents/g of sample, HU/mg: Hemolysis units/mg of sample. Results represent the mean ± standard deviation (SD) values of triplicate experiments.

## Data Availability

The original contributions presented in this study are included in the article. Further inquiries can be directed to the corresponding authors.

## Abbreviations

The following abbreviations are used in this manuscript:

BAPNA	N-benzoyl-DL-arginine p-nitroanilide
CE	Catechin equivalents
DAU	Daunorubicin
FBS	Fetal bovine serum
HAU	Hemagglutination activity units
HU	Hemolytic units
IC_50_	Half-maximal inhibitory concentrations
LD_50_	Median lethal dose
MN	Micronucleus
MNPE	Micronucleated polychromatic erythrocytes
NE	Normochromatic erythrocytes
PBS	Phosphate-buffered saline
PE	Polychromatic erythrocytes
SD	Standard deviation
TBE	Tepary bean protein extract
TIU	Trypsin inhibitor units
